# Autophagy inhibitor chloroquine increases sensitivity to cisplatin in QBC939 cholangiocarcinoma cells by mitochondrial ROS

**DOI:** 10.1371/journal.pone.0173712

**Published:** 2017-03-16

**Authors:** Xianzhi Qu, Jiyao Sheng, Luyan Shen, Jing Su, Yunjie Xu, Qi Xie, Yao Wu, Xuewen Zhang, Liankun Sun

**Affiliations:** 1 Department of Hepatobiliary & Pancreat Surgery, The Second Hospital of Jilin University, Jilin University, Changchun, Jilin, China; 2 Department of Pathophysiology, School of Basic Medical Sciences, Jilin University, Changchun, Jilin, China; University of Nebraska-Lincoln, UNITED STATES

## Abstract

The tumor cells have some metabolic characteristics of the original tissues, and the metabolism of the tumor cells is closely related to autophagy. However, the mechanism of autophagy and metabolism in chemotherapeutic drug resistance is still poorly understood. In this study, we investigated the role and mechanism of autophagy and glucose metabolism in chemotherapeutic drug resistance by using cholangiocarcinoma QBC939 cells with primary cisplatin resistance and hepatocellular carcinoma HepG2 cells. We found that QBC939 cells with cisplatin resistance had a higher capacity for glucose uptake, consumption, and lactic acid generation, and higher activity of the pentose phosphate pathway compared with HepG2 cells, and the activity of PPP was further increased after cisplatin treatment in QBC939 cells. It is suggested that there are some differences in the metabolism of glucose in hepatocellular carcinoma and cholangiocarcinoma cells, and the activation of PPP pathway may be related to the drug resistance. Through the detection of autophagy substrates p62 and LC3, found that QBC939 cells have a higher flow of autophagy, autophagy inhibitor chloroquine can significantly increase the sensitivity of cisplatin in cholangiocarcinoma cells compared with hepatocellular carcinoma HepG2 cells. The mechanism may be related to the inhibition of QBC939 cells with higher activity of the PPP, the key enzyme G6PDH, which reduces the antioxidant capacity of cells and increases intracellular ROS, especially mitochondrial ROS. Therefore, we hypothesized that autophagy and the oxidative stress resistance mediated by glucose metabolism may be one of the causes of cisplatin resistance in cholangiocarcinoma cells. It is suggested that according to the metabolism characteristics of tumor cells, inhibition of autophagy lysosome pathway with chloroquine may be a new route for therapeutic agents against cholangiocarcinoma.

## Introduction

Hepatocellular carcinoma and cholangiocarcinoma are the two most common primary tumors in the hepatobiliary system. They have different metabolic characteristics due to their different tissue sources[1]. Compared with hepatocellular carcinoma (HCC), cholangiocarcinoma (CC) cells have primary resistance to chemotherapeutic drugs such as cisplatin [2, 3]. In addition, it has been found that reduction–oxidation (REDOX) signaling pathways play a major role in cancer formation and especially in responses to radiotherapy and chemotherapy. Thus, many researchers are focusing on induction of oxidative stress for anti-tumor therapy. Cells generate reactive oxygen species (ROS) through the processes of metabolism, respiratory burst, and the respiratory chain, and clear ROS via the peroxisome, SOD, NADPH-dependent reduction system, and autophagy-lysosome pathway to regulate the REDOX balance in cells. In the REDOX balance adjustment process, the main source of mitochondrial (mt) ROS is oxidative respiration. Disruption of mitochondrial functions can also increase mtROS production and induce cell death [4, 5]. Chemotherapeutic drugs such as cisplatin can combine with mitochondrial DNA, disrupt mitochondrial functions, increase mtROS [6], and induce cell death. In addition, cells generating mtROS can further induce mitochondria to produce more ROS and increase the cellular REDOX imbalance [6, 7]. Therefore, mtROS is considered to be an important indicator of the REDOX balance [7], and elevating mtROS may be an effective approach for cancer therapy.

Glucose metabolism can regulate cell redox balance [8]. Because of the metabolism in tumor cells, the oxidative stress level is high, and the metabolic antioxidant capacity also increases to maintain their survival, leading to strong balancing of oxidation and anti-oxidation [9]. Tumor cell metabolism of glucose in the aerobic glycolysis (Warburg’s effect) shift, ensures the required energy and provides biosynthesis of macromolecules, which can reduce the oxidative stress level of tumor cells and promote their proliferation. A previous study has reported that an increase in aerobic glycolysis may induce the pentose phosphate pathway (PPP), another branch of key glucose metabolism [10]. The PPP provides more than 60% of the NADPH in cells. Studies in multiple myeloma cells and MCF-7 human breast cancer cells showed enhancement of the PPP, and that PPP-derived NADPH enhances the cellular antioxidant capacity, mediating resistance to epidermal growth factor receptor-targeted drugs and adriamycin [11, 12]. Trans-dehydroandrosterone (DHEA), a non-competitive antagonist of the PPP key enzyme glucose-6-phosphate dehydrogenase (G6PDH), reduces NADPH levels, resulting in a lack of substrate to maintain the reduction status of glutathione (GSH), which reduces the resistance of tumor cells to oxidative stress [13] and increases the tumor cell sensitivity to chemotherapeutic drugs. Therefore, suppressing the PPP might weaken the antioxidant capacity of cells. It has been speculated that cell metabolism-mediated oxidative stress resistance may be associated with drug resistance in tumor cells, and changing the metabolism-related antioxidant capacity may inhibit tumor cell proliferation. However, the REDOX balance within tumor cells has many redundant regulatory mechanisms, and inhibition of glucose metabolism alone may not suppress tumors. Therefore, we examined the characteristics of tumor cell metabolism, the REDOX state, and inhibiting the anti-oxidation ability of tumor cells to provide a basis for individualized treatment of cancer.

Mitochondria are a major source of oxidative stress in cells, and the REDOX balance has a close relationship with cell survival. MtROS in the respiratory chain is the main source of oxidation, and ROS can be removed by many pathways. In the mitochondrial matrix, the SOD protein family, GSH/GSSG (glutathione disulfide), and the thioredoxin reduction system are the main routes for internal anti-oxidation, which rely on metabolites of NADPH for antioxidant effects. When mtROS exceeds its own adjustment ability, mitochondrial permeability transition pores (mPTPs) open and mtROS are released into the cytoplasm and eliminated by ROS removal mechanisms such as the peroxisome and autophagy-lysosomal pathway [7]. Dysfunctional mitochondria can also be removed by mitochondrial autophagy degradation [14]. High levels of mtROS need to be removed through the various antioxidant mechanisms of which the autophagy-lysosome pathway plays a very important role in common regulation of metabolic substrates and ROS in cells.

Autophagy is necessary for the maintenance of cellular metabolism and activated by mitochondrial dysfunction and oxidative stress. In addition to increasing the antioxidant capacity, cells can increase the degradation of damaged proteins and organelles through the autophagy-lysosome pathway to maintain cellular metabolism and the mitochondrial REDOX state [15]. Classic autophagy inhibitors 3-methyladenine (3-MA) and chloroquine (CQ) can inhibit lung and colon cancers and thus have potential for tumor treatment [16–18]. The small alkaline molecule CQ can accumulate in lysosomes, which will change the lysosome acidity, causing reduced hydrolysis. It can also cause an increase in lysosomal membrane permeability, releasing small molecules and ions, including Fe^2+^, into the cytoplasm together with H_2_O_2_ causing Fenton’s reaction to generate hydroxyl free radicals, leading to elevated ROS levels in cells. Ganguli *et al*. found that treatment of A549 cells with 50 μM CQ decreases the mitochondrial membrane potential, indicating that CQ causes mitochondrial damage that leads to increased intracellular ROS [19]. Seung *et al*. used 10 μM CQ to examine for mechanisms of the additive effects of temozolomide and autophagy inhibitors in glioma cells [20]. Inhibition of autophagy, which may interfere with glucose metabolism, reduces the efficiency of intracellular ROS removal and the oxidation reduction functions of lysosomes to further increase mtROS production. These effects suggest that CQ inhibition of autophagy lysosome pathway may have a potential effect on tumor inhibition.

Autophagy changes the REDOX equilibrium. Cells with disrupted autophagy functions show a decrease in glucose uptake and glycolysis flux [21], and inhibition of the glucose metabolism-related antioxidant capacity. Studies have shown that autophagy-enhancing drugs increase the tolerance of tumor to mitochondrial damage [22–24]. In addition, another study reported that interference of autophagy by increasing the lysosome pH leads to the release of iron from lysosomes and redistribution in the cytoplasm, inducing Fenton’s reaction and increasing the oxidative stress level in cells [25].

In summary, various ways to enhance the antioxidant ability of tumor cells is closely related to the tumor drug resistance, Inhibition of autophagy may reduce the antioxidant capacity of drug resistant cells by inhibiting a variety of ways, including glucose metabolism, and increase the sensitivity of cisplatin induced death. Therefore, in this experiment, we chose two different kinds of hepatobiliary tumor, hepatocellular carcinoma HepG2 cells and cholangiocarcinoma QBC939 cells, to compared differences in cisplatin resistance, metabolism and antioxidant capacity, and to investigate the role of autophagy inhibitor CQ in increasing the level of intracellular mtROS by inhibiting the PPP and promoting the production of intracellular ROS, and further enhancing cisplatin induced QBC939 cells death. Such a strategy may provide a new approach for drug-resistance reversion by exploring the mechanism of reducing the activity of glucose metabolism through inhibiting autophagy-lysosomal pathway to increase the sensitivity of tumor cells to cisplatin.

## Materials and methods

### Cell cultures and reagents

The human HCC line HepG2 and CC cell line QBC939 were obtained from the cell bank of the Institute of Biochemistry and Cell Biology (Shanghai, China) and the Third Military Medical University, respectively. Both cell types were cultured in RPMI-1640 medium (Gibco, Carlsbad, CA), supplemented with 10% fetal bovine serum (FBS; HyClone, Logan, UT) in a 5% CO_2_ humidified environment at 37°C. RIPA lysis buffer, Trypan Blue and JC-1 was from Beyotime (Shanghai, China). Antibodies used were rabbit polyclonal p62 and anti-LC3 (1:1,000) from Abcam, anti-G6PDH (1:200) from Santa Cruz Biotechnology (Santa Cruz, CA), and mouse monoclonal anti-β-actin (1:1,000) and horseradish peroxidase-conjugated secondary antibodies (1:1,000) from Proteintech (Chicago, IL). MitoSOX Red mitochondrial superoxide indicator was purchased from Invitrogen (Carlsbad, CA). 2’,7’-Dichlorofluorescin diacetate (DCFH-DA), 3-(4,5-dimethylthiazol-2-yl)-2,5-diphenyltetrazolium bromide (MTT), 3-Methyladenine (3-MA), (2-(2,2,6,6-Tetramethylpiperidin-1-oxyl-4-ylamino)-2-oxoethyl)triphenylphosphonium chloride (Mito-TEMPO), chloroquine diphosphate salt (CQ), cisplatin, and 2-deoxy-2-[(7-nitro-2,1,3-benzoxadiazol-4-yl)amino]-D-glucose (2-NBDG) were from Sigma-Aldrich (St. Louis, MO). 2-DG and DHEA was purchased from Aladdin industrial Corporation (Shanghai, China).

### Cell viability and cell death

Cell viability and cell death were measured using the MTT assay and Trypan Blue assay, respectively. For MTT assays, each treatment was replicated in four wells, 10 μl 5 mg/ml MTT was added to each well and after incubation for 4 h the formazan crystals were dissolved with 150 μl dimethyl sulfoxide. Absorbance was measured at a wavelength of 570 nm using a microplate reader (Molecular Devices, Sunnyvale, CA). For Trypan Blue assay, each treatment was replicated in three wells. Detach live cells by incubating with 0.4% trypsin, making sure to keep any washes. Add the cells in medium plus the cells from any wash steps to the detached cells. Cells concentrated by centrifugation (500g for 5 min) and resuspended with 200 μl. Mix 50 μL of each sample cells with an equal volume of 0.4% trypan blue solution. Add 10 μL of the sample to the hemocytometer under the coverslip. Observe trypan blue-stained cells using a standard bright-field microscope. Count at least 100 cells across all four fields of the hemocytometer, including blue (dead) and colorless (healthy) cells. Each sample repeat three times at least. Calculate the percentage of dead cells using the following equation: %Cell death = Number of colorless cells ÷ Total number of cells × 100.

### Analysis of glycolysis

After cell attachment the medium was replaced with fresh 10% FBS RPMI-1640 with or without 20 μg/ml cisplatin and the cells were allowed to grow for 6 h. Medium was collected from the plates, centrifuged at 100 g for 5 min, aliquoted, and frozen at −80°C. Samples were thawed and diluted for analysis of metabolites. Glucose consumption and L-lactate production were measured using commercial colorimetric kits according to the manufacturer's protocol. The glucose assay kit was from RsBio (Shanghai, China) and the L-lactate kit was from Jiancheng Bio (Nanjing, China). The metabolite consumption and production values were normalized to the corresponding total amount of protein in each plate as determined by the Bio-Rad protein assay (Bio-Rad, Hercules, CA) performed on cell lysates.

### Analysis of the pentose-phosphate pathway

The medium was replaced with fresh 10% FBS RPMI-1640 with or without 50 μM CQ or 5 mM 3-MA or 20 μg/ml cisplatin, and the cells were allowed to grow for a further 6 h. After harvesting, 1×10^6^ cells were collected and washed with PBS three times. Glucose-6-phosphate dehydrogenase activity, NADPH/NADP ratio, and GSH/GSSG ratio were measured using commercial colorimetric kits according to the manufacturer's protocol. The glucose-6-phosphate dehydrogenase assay kit was from Sigma-Aldrich, the NADP/NADPH quantification colorimetric kit was from BioVision (San Francisco, CA), and the total glutathione/oxidized glutathione assay kit was from Jiancheng Bio.

### Western blot analysis

After drug treatment for 24 h, QBC939 cells and HepG2 cells were harvested, washed with cold PBS, and incubated in RIPA lysis buffer for 40 min at 4°C to isolate total proteins. The concentration of total proteins was measured using the Bio-Rad assay. Protein samples (30 μg for each group) were separated by 12% SDS-PAGE and transferred to PVDF membranes (Roche, Basel, Switzerland), which were blocked with buffer (100 mM NaCl, 10 mM Tris–HCl [pH7.6], and 0.1% Tween 20) containing 5% non-fat dry milk for 1.5 h at room temperature. Membranes were incubated with various primary antibodies overnight at 4°C and with the corresponding secondary antibodies at room temperature for 1.5 h. The bands were visualized using Pierce ECL Western Blot Substrate (Thermo Scientific, Waltham, MA).

### Flow cytometry

Cells were exposed to 20 mg/ml cisplatin and/or 50 μM CQ or 5 mM 3-MA for 8 or 12 h, trypsinized, and stained with Annexin V-FITC and propidium iodide (Annexin V Apoptosis Detection Kit, BestBio, Shanghai, China) to measure cellular apoptosis. Untreated cells were trypsinized and exposed to 50 μM 2-NBDG (Sigma-Aldrich) in glucose-free and serum-free DMEM for 30 min to evaluate the glucose uptake capacity. JC-1 (Beyotime Biotechnology, Shanghai, China) was used to evaluate MMP. Analysis was performed using a BD Accuri C6 flow cytometer (Becton Dickinson, Franklin Lakes, NJ).

### Fluorescent staining

The production of total ROS and mtROS was evaluated by staining with DCFH-DA and MitoSOX, respectively. Cells were then incubated with 10 mM DCFH-DA for 30 min at 37°C or with 5 μM MitoSOX for 10 min at 37°C. After washing with PBS, the samples were observed using an IX71 fluorescence microscope (Olympus, Japan). Quantitation of the cell average fluorescence intensity was processed by software Image J (version 2.1.4.7), and more than 200 cells of each sample were analyzed.

### Statistical analysis

Statistical analysis of the data was performed using one-way ANOVA. All experiments were repeated at least three times and presented as the mean±standard error (SE). The Student’s t-test was used to analyze differences between the values of two samples. *p*<0.05 was considered statistically significant.

## Results

### Cisplatin resistance in QBC939 CC cells

**Fig 1:**
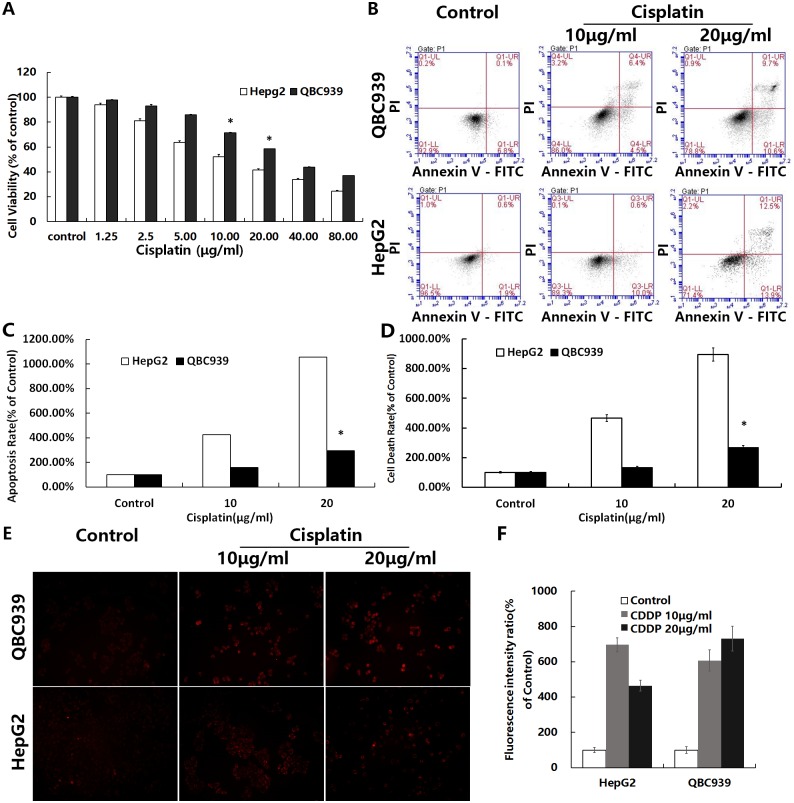
QBC939 cells are resistant to cisplatin. QBC939 and HepG2 cells were treated with cisplatin for 12 or 24 h, and then cell viability, apoptosis and cell death rates, and mtROS were measured. (A) Cells were treated with cisplatin (1.25–80 μg/ml) for 24 h and then cell viability was determined by MTT assays. (B) Cells were treated with cisplatin (10 or 20 μg/ml) for 12 h and then the apoptotic rate was measured by flow cytometry (fluorescence intensity: x axis, Annexin V-FITC; y axis, PI). (C) Quantitation of the apoptosis rate (including early and late apoptosis) under the same treatment conditions as in (B). (D) Quantitation of the cell death rate by trypan blue staining under the same treatment conditions as in (B). (E) Cells were treated with cisplatin (20 μg/ml) for 24 h followed by 5 μm mitoSOX for 10 min, and then fluorescence intensity was observed by fluorescence microscopy (×200). (F) Quantitation of the cell average fluorescence intensity under the same treatment conditions as in (E). All values are the mean±SE. *p<0.05 between QBC939 cells group and HepG2 cells group.

We measured the viability, apoptosis, and cell death rates of QBC939 cells treated with cisplatin for 12 or 24 h (Fig 1A–1D). The percentages of apoptosis and overall cell death showed that the type of cisplatin induced cell death was mainly apoptotic cell death and that QBC939 cells exhibited greater resistance to cisplatin than HepG2 HCC cells.

### MtROS generation and QBC939 cells resistance to cisplatin

Previous studies have reported that cisplatin increases the levels of intracellular ROS such as mtROS [6]. To observe changes in mtROS of QBC939 cells treated with cisplatin, we used the mtROS-specific fluorescent probe MitoSOX. Cells showed an increase of mtROS induced by cisplatin. QBC939 cells showed a small increase in mtROS, which induced strong resistance to cisplatin (Fig 1E and 1F).

**Fig 2:**
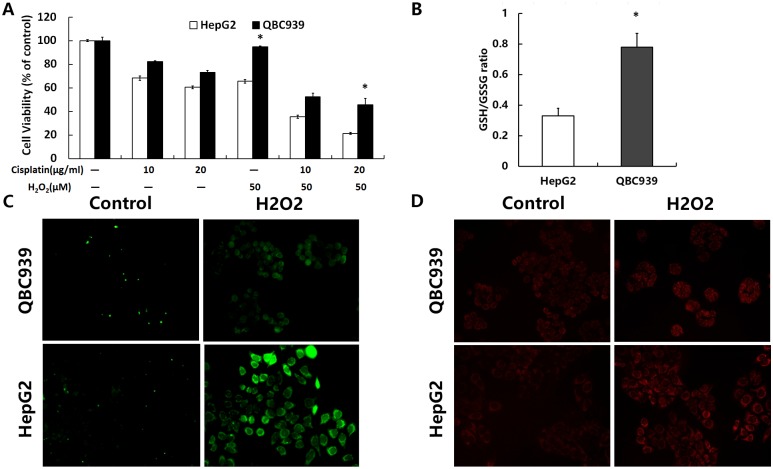
The antioxidant capacity of QBC939 cells were stronger than HepG2 cells. (A) Cells were treated with H_2_O_2_ (50 μM) or cisplatin (10 or 20 μg/ml) for 24 h, and then cell viability was determined by MTT assays. (B) The intracellular GSH/GSSG ratio was determined after incubation under basal conditions. (C) Overall ROS and (D) mtROS were measured in QBC939 cells and HepG2 cells treated with 500 μM H_2_O_2_ for 2 h (×200). All values are the mean±SE. *p<0.05 between QBC939 cells group and HepG2 cells group.

After treatment with various concentrations of H_2_O_2_ for 24 h, we detected cell viability (Fig 2A). The results suggested that the capacity of exogenous ROS resistance in QBC939 cells was more than that in HepG2 cells, and impaired the antioxidant capacity of cells with H_2_O_2_ increases sensitivity to cisplatin. Reducing glutathione is an important antioxidant system, and the intracellular GSH/GSSG ratio presumably indicates a cell’s anti-oxidation ability. Next, we analyzed the intracellular GSH/GSSG antioxidant system (Fig 2B). The basal level of GSH/GSSG in QBC939 cells was significantly higher than that in HepG2 cells. DCFH-DA can be evaluated by its green fluorescence when combined with intracellular ROS components. Thus, the brightness of the fluorescence is proportional to the overall amount of ROS in cells. Compared with HepG2 cells, the overall ROS and mtROS increase was less in QBC939 cells treated with H_2_O_2_ (Fig 2C and 2D), indicating that QBC939 cells have a stronger antioxidant ability.

### QBC939 cells uptake more glucose and generate more lactic acid

**Fig 3:**
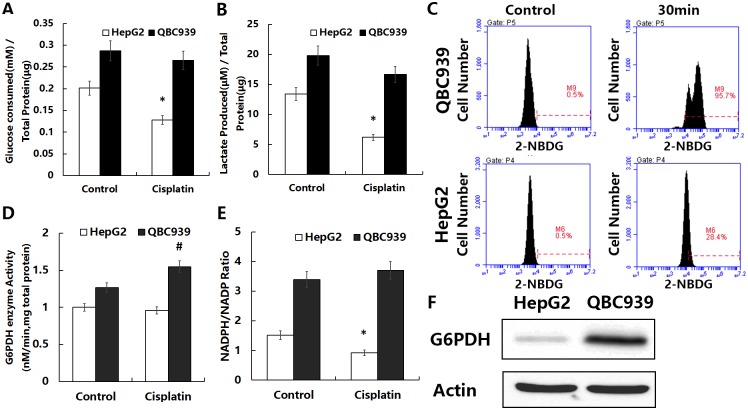
Enhancement of the glucose metabolism-derived antioxidant capacity in QBC939 cells. (A) Consumption of glucose in the medium, (B) production of lactic acid, (D) activity of G6PDH, and (E) ratio of NADPH/NADP after incubation under basal conditions or treated with cisplatin (20 μg/ml) for 6 h. (C) Fluorescence intensity changes were measured after incubation for 30 min with or without 50 μM 2-NBDG using flow cytometry (x axis, fluorescence intensity; y axis, cell number). (F) Immunoblot analysis of G6PDH expression under basal conditions. All values are the mean±SE. *p<0.05 between control and other group in QBC939 cells, ^#^*p*<0.05 between control and other group in HepG2 cells.

To investigate the metabolic characteristics of QBC939 cells, we tested their glucose metabolism performance. Venmar *et al*. measured cellular glucose uptake and changes in lactic acid secretion to evaluate changes in cell glucose metabolism [26]. We measured the amount of glucose consumption and lactic acid secretion after 6 h under basic condition or treated with cisplatin in QBC939 cells compared with HepG2 cells (Fig 3A and 3B). The mean values for glucose consumption and lactic acid production of QBC939 cells were significantly higher under basic condition and decreased less after cisplatin treatment than those of HepG2 cells. Treated cells with a fluorescent glucose analog substrate (2-NBDG) allow direct observation of the glucose uptake capacity [27]. Therefore, we treated QBC939 and HepG2 cells with 2-NBDG and used flow cytometry to detect fluorescence changes in the cells before and after 30 min of treatment. As shown in Fig 3C, the glucose uptake efficiency of QBC939 cells was higher than that of HepG2 cells. These results suggested that QBC939 cells have a more efficient glucose metabolism.

### QBC939 cells have a more active PPP

The PPP is a bypass pathway for glucose metabolism and an important source of NADPH in cells. The first step in the pathway is the rate-limiting reaction of oxidation of NADP to NADPH by G6PDH. G6PDH is therefore a key enzyme, and its activity determines the efficiency of the PPP [28]. Moreover, increases in the intracellular NADPH/NADP ratio can reflect PPP efficiency. Therefore, we measured the expression level under basic condition and activity of G6PDH and the NADPH/NADP ratio under basic condition or treated with cisplatin in both two kinds of cells to determine the metabolic efficiency of the PPP (Fig 3D–3F). The G6PDH expression and activity as well as the NADPH/NADP ratio were higher under basic condition in HepG2 cells. Contrary to HepG2 cells, the G6PDH activity and the NADPH/NADP ratio were increased after cisplatin treatment in QBC939 cells. These results above suggested that QBC939 cells may have a more efficient PPP, and treatment with cisplatin may enhance the activity of PPP in QBC939 cells.

### Glucose metabolism inhibitors increase ROS levels in QBC939 cells

**Fig 4:**
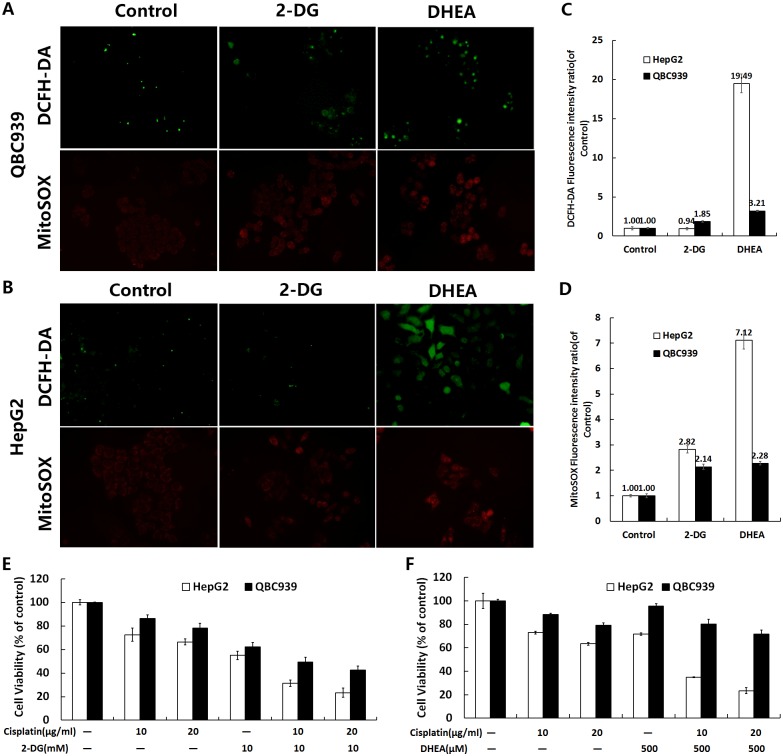
Inhibition of glucose metabolism reduces the antioxidant capacity of QBC939 cells. Cells were treated with 20 mM 2-DG and 500 μM DHEA for 24 h and assayed for changes in the level of overall ROS and mtROS in (A) QBC939 cells and (B) HepG2 cells (×200). Quantitation of the cell average fluorescence intensity of (C) DCFH-DA and (D) MitoSOX under the same treatment conditions as in (A) and (B). Cells were treated with (E) 10 mM 2-DG or (F) 500 μM DHEA, alone or with the combination of cisplatin (10 or 20 μg/ml) respectively, for 24 h and then analyzed by MTT assays to detect cell viability. All values are the mean±SE.

2-DG and DHEA inhibit glucose metabolism by inhibiting hexokinase 2 and G6PDH, respectively [13, 29]. We tested the sensitivity of QBC939 cells to 2-DG or DHEA and/or combined with cisplatin (Fig 4E and 4F). Compared with HepG2 cells, QBC939 cells were less sensitive to DHEA. We measured overall intracellular ROS and mtROS after treatment with 2-DG and DHEA. As shown in Fig 4A–4D, both metabolic inhibitors increased ROS production, indicating that an increased rate of ROS production was associated with cellular sensitivity to these drugs. Therefore, glucose metabolism inhibitors reduce the antioxidant capacity in QBC939 cells and increase ROS levels.

### Sensitivity of the autophagy inhibitor CQ and 3-MA in QBC939 cells

**Fig 5:**
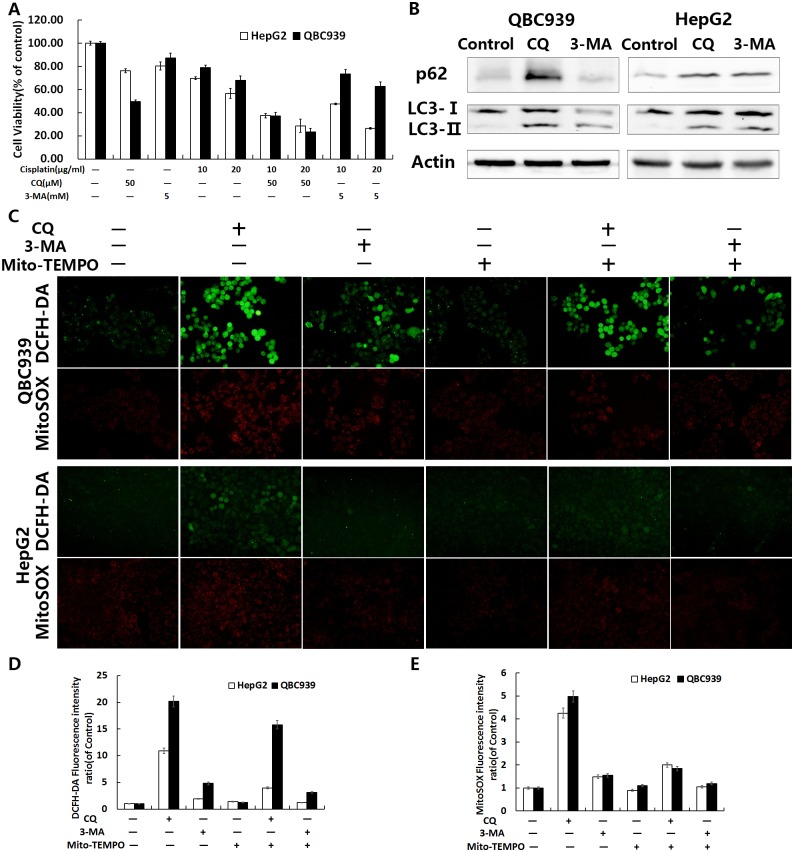
CQ inhibited autophagy and enhanced ROS in QBC939 cells. (A) Cells were treated with CQ (50 μM) or 3-MA (5 mM) and/or cisplatin (10 or 20 μg/ml) for 24 h and then cell viability was measured by MTT assays. (B) Immunoblot analysis of intracellular p62 and LC3-II/I in cells treated with CQ (50 μM) or 3-MA (5 mM) for 24 h. Overall ROS and mtROS were measured in (C) QBC939 cells and HepG2 cells treated with 100 μM Mito-TEMPO and/or CQ (50 μM) or 3-MA (5mM) for 12 h (×200). Quantitation of the cell average fluorescence intensity of (D) DCFH-DA and (E) MitoSOX under the same treatment conditions as in (C). All values are the mean±SE.

Because autophagy is closely related to the intracellular antioxidant capacity [30], and CQ and 3-MA can effectively inhibit autophagy, we speculated that CQ and 3-MA would have a potential antitumor effect. We therefore tested QBC939 cells for the sensitivity of CQ or 3-MA and/or combined with cisplatin as shown in Fig 5A. Compared with HepG2 cells, CQ significantly inhibited QBC939 cell viability, indicating that QBC939 cells are more sensitive to lysosome inhibition. After 24 h of CQ or 3-MA treatment, the cells were analyzed by western blotting to detect intracellular p62 and LC3-II/I. The levels of p62 and LC3-II/I were increased significantly after CQ treatment in QBC939 cells (Fig 5B), suggesting that QBC939 cells have a higher flow of autophagy.

### Mito-TEMPO could inhibit the increase of ROS in QBC939 cells induced by CQ

MitoTEMPO is a mitochondrially targeted antioxidant, a specific scavenger of mitochondrial superoxide. After treatment with CQ or 3-MA alone and/or combined with Mito-TEMPO, we detected the intracellular ROS of both cell lines. We found that Mito-TEMPO significantly inhibited CQ induced intracellular overall ROS and mtROS, as shown in Fig 5C–5E, especially mtROS levels.

### Treatment with CQ increased cisplatin induced mtROS in QBC939 cells

**Fig 6:**
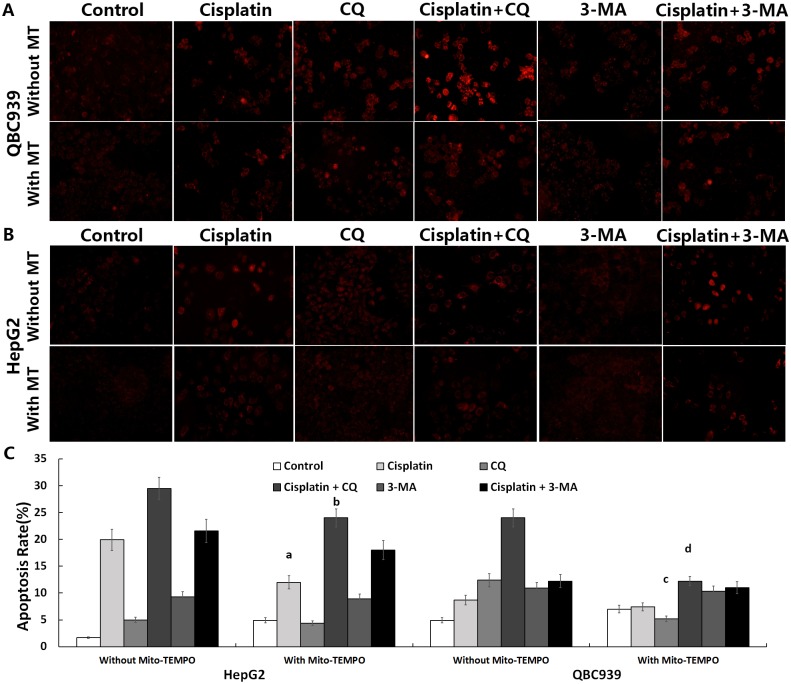
CQ enhanced cisplatin-induced apoptosis in QBC939 cells. (A) QBC939 cells and (B) HepG2 cells were treated with CQ (50 μM) or 3-MA (5 mM) and/or cisplatin (20 μg/ml) and/or Mito-TEMPO (100 μM) for 12 h or 8 h and then assayed for the level of mtROS (×200) and apoptosis rate was measured by flow cytometry, respectively. Quantitation of apoptosis rate (including early and late apoptosis) was shown in (C). All values are the mean±SE. ^a,b^*p*<0.05 between with and without Mito-TEMPO group in HepG2 cells, ^c,d^*p*<0.05 between with and without Mito-TEMPO group in QBC939 cells.

After treatment with CQ, 3-MA alone or combined with cisplatin and/or Mito-TEMPO, we observed mtROS fluorescence staining in cells. As shown in Fig 6A and 6B, the levels of mtROS of CQ combined with cisplatin were increased much higher than that of CQ alone in QBC939 cells. After the treatment combined with Mito-TEMPO, mtROS levels were reduced significantly.

After treatment with CQ, 3-MA alone or combined with cisplatin and/or Mito-TEMPO, we detected the sensitivity to cisplatin-induced apoptosis by flow cytometry. The apoptosis rate of CQ combined with cisplatin was significantly higher than that of cisplatin alone (Fig 6C), indicating that CQ increased sensitivity to cisplatin-induced apoptosis. By analyzing the apoptosis rate of combination with Mito-TEMPO, we found that Mito-TEMPO significantly decreased the sensitivity to apoptosis induced by CQ alone or combination with cisplatin (Fig 6C). It is suggested that the increase of sensitivity to CQ and cisplatin induced apoptosis may be related to the increase of mtROS in QBC939 cells, and inhibition of mtROS restrains sensitivity to apoptosis in QBC939 cells treated with CQ and cisplatin.

### CQ inhibits PPP activity and antioxidant capacity in QBC939 cells

**Fig 7:**
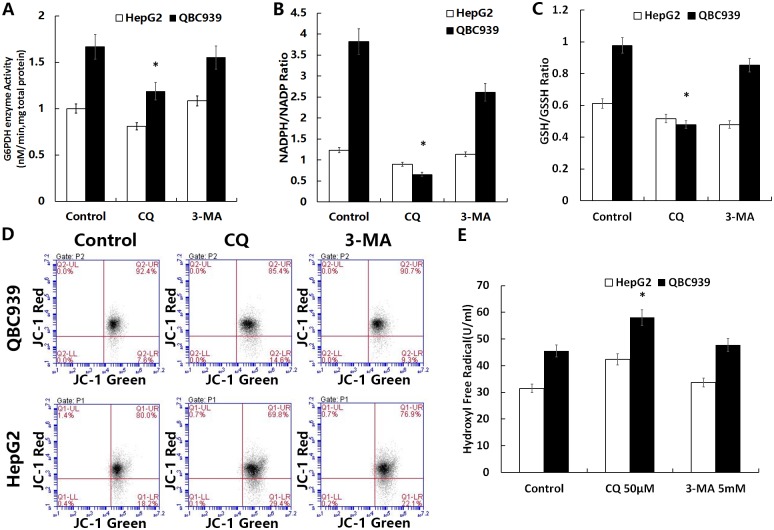
CQ reduced the glucose metabolism-related antioxidant capacity and mitochondrial membrane potential in QBC939 cells and increases the amount of intracellular hydroxyl free radicals. Cells were treated with CQ (50 μM) or 3-MA (5 mM) for 6 h and then assayed for (A) intracellular G6PDH activity, (B) the NADPH/NADP ratio, and (C) GSH/GSSG ratio. (D) Cells treated with CQ (50 μM) or 3-MA (5 mM) were incubated for 8 h and then assayed for the mitochondrial membrane potential with JC-1 by flow cytometry (fluorescence intensity: x axis, green; y axis, red). (E) Cells treated with CQ (50 μM) or 3-MA (5 mM) were incubated for 24 h and then assayed for the amount of intracellular hydroxyl free radicals. All values are the mean±SE. *p<0.05 between control and other group in QBC939 cells.

To determine whether the increased production of ROS was associated with inhibition of the glucose metabolism-related antioxidant capacity, we measured G6PDH activities, the NADPH/NADP ratio, and GSH/GSSG ratio in QBC939 cells treated with CQ and 3-MA for 6 h. After treatment with CQ, significant reductions in all three parameters were found in QBC939 cells (Fig 7A–7C). We speculated that suppressing QBC939 cell autophagy-lysosomal pathway inhibits their glucose metabolism-related antioxidant capacity.

### CQ inhibits mitochondrial function of QBC939 cells

By testing the mitochondrial membrane potential using JC-1, we found decreases of mitochondrial membrane potentials in both two kinds of cells by CQ or 3-MA treatment for 8 h (Fig 7D). These results showed that increasing mtROS may induce opening of mPTPs, leading to a lower membrane potential.

### CQ increases hydroxyl free radicals produced in QBC939 cells

CQ can easily accumulate in a low pH environment, such as in lysosomes, increasing membrane permeability and releasing metal ions such as Fe^2+^ into the cytoplasm where Fenton’s reaction occurs with H_2_O_2_, generating a large amount of hydroxyl free radicals. We detected the quantity of hydroxyl free radicals in cells, and found that hydroxyl free radicals were increased significantly after treatment with CQ for 24 h (Fig 7E). Therefore, after inhibition of autophagy with CQ, the overall ROS in QBC939 cells increased, which might be related to the increase of hydroxyl radical production.

## Discussion

Galluzzi *et al*. previously reported that cisplatin increases intracellular ROS levels [6]. Tumor drug resistance is closely related to their antioxidant capacity [12, 31], and the antioxidant capacity of tumor cells is also closely related to their metabolism. Early studies reported that CC cells from the liver had primary cisplatin resistance [2, 3]. Our study indicated that the sensitivity of QBC939 CC cells, which are derived from the liver bile duct epithelium, to cisplatin was significantly lower than that of HepG2 HCC cells (Fig 1A–1D). The fluorescence intensity of staining for mtROS and the GSH/GSSG ratio were significantly higher in QBC939 cells than in HepG2 cells (Figs 1E, 1F and 2B). Cells treated with cisplatin for 24 h showed a significant increase in mtROS, especially cisplatin-sensitive HepG2 cells compared with cisplatin-resistant QBC939 cells (Fig 1E and 1F). QBC939 cells had lower susceptibility to H_2_O_2_ (Fig 2A, 2C and 2D), indicating strong antioxidant capacity in CC cells, which might explain their primary cisplatin resistance.

It is currently thought that glucose metabolic pathways regulate the cellular REDOX state [8]. We found that glucose consumption and lactic acid production in QBC939 cells were higher than those in HepG2 cells (Fig 3A–3C), indicating that these CC cells are more dependent on glucose metabolism. Studies have reported that proto-oncogene-induced cancer cells have altered antioxidant pathways and regulatory factors and that tumor cells with higher glucose metabolism have higher oxidation levels and enhanced oxidation resistance through other pathways [10, 32, 33]. These properties maintain the REDOX balance at a higher level, thus promoting tumor proliferation or escape from cell death. Our study also showed that the activity and expression of the key PPP enzyme G6PDH and the NADPH/NADP ratio were higher in QBC939 cells than in HepG2 cells (Fig 3D–3F). We speculate that the efficiency of the PPP is higher in QBC939 cells than in HepG2 cells. Studies have shown that oxidation of the PPP can be activated within a few seconds in human skin cells treated with H_2_O_2_, producing more NADPH and enhancing the capacity for oxidation reduction [34]. Our study showed that the sensitivity of QBC939 cells to H_2_O_2_ was significantly lower than that of HepG2 cells (Fig 2A, 2C and 2D). Consequently, we hypothesize that the increased glucose metabolism in QBC939 cells increases their antioxidant capacity, which is related to an enhanced PPP. After the treatment with cisplatin, the glucose consumption and lactic acid production of QBC939 cells were decreased less than those of HepG2 cells, and on the contrary to HepG2 cells, the G6PDH activity and the NADPH/NADP ratio were increased after cisplatin treatment in QBC939 cells. Thus, the cisplatin resistance of CC cells may be mediated by the glucose metabolism-related antioxidant capacity.

Previous studies have suggested that inhibiting glucose metabolism can increase ROS levels in tumor cells and inhibit tumor proliferation. Sinthupibulyakit *et al*. reported that the glucose analog 2-DG increases mtROS levels in lung cancer cells [35]. Our cell viability experiments showed that QBC939 cells had the similar sensitivity in 2-DG with HepG2 cells, and that the levels of overall ROS and mtROS were increased (Fig 4A–4D). Inhibition of the PPP might further increase intracellular ROS levels and inhibit tumor growth. Indeed, we found that DHEA increased overall ROS and mtROS levels in HepG2 cells (Fig 4A–4D), which were significantly higher than those in QBC939 cells, suggesting that inhibition of the PPP can increase cellular oxidative stress. However, QBC939 cells had low sensitivity to DHEA (Fig 4E and 4F), which may be related to their high antioxidant capacity. Therefore, inhibition of the PPP might be insufficient to cause ROS-induced cell death in CC cells alone.

Some recent research found that the degradation of damaged proteins and organelles by autophagy, a lysosome-mediated pathway, is the main method to maintain normal metabolism and the REDOX state in cells [15, 25]. Desideri *et al*. reported that, under starvation conditions, ROS levels in HeLa cells increase and activation of AMPK induces autophagy. Hydrolysis components provide substrates for cell metabolism, remove oxidized and abnormally functioning proteins and organelles such as damaged mitochondria, and increase the capability for ROS removal [36]. These observations suggest that autophagy is closely related to the maintenance of intracellular REDOX equilibrium. In addition to the degradation of damaged organelles and oxidized proteins, autophagy plays an antioxidant role by activation of Nrf2 [37]. Inhibition of autophagy in human U251 glioma cells with 3-MA increases their sensitivity to apoptosis induced by H_2_O_2_ [30]. Based on these metabolic characteristics, inhibition of autophagy may increase mtROS production or reduce ROS removal, thus enhancing the level of ROS and either killing tumor cells or inhibiting their proliferation.

Hou *et al*. reported that 3-MA inhibits early autophagy activation processes, induces CC cell apoptosis during metabolic stress, and increases drug sensitivity [38]. CQ is thought to be dissimilar to the autophagy initiation type III PI3K inhibitor 3-MA because it inhibits the function of lysosomes, resulting in a widespread suppression of autophagy. Oxidized proteins cannot be degraded by molecular chaperone-mediated autophagy in lysosomes, which are inhibited by CQ. Therefore, CQ is likely to have a stronger inhibitory effect on the antioxidant capacity and cell-death-inducing effects compared with 3-MA [39]. We compared the effects of the autophagy inhibitor CQ and 3-MA with or without the combination of cisplatin on QBC939 and HepG2 cells, which have different metabolic characteristics and REDOX states. We found that CQ inhibited QBC939 cell viability much more than that of HepG2 cell, and after treated with the combination of CQ and cisplatin, the cell viability of QBC939 cell was further reduced than cisplatin alone (Fig 5A). Our data further suggested that QBC939 cell may be more dependent on the lysosomal degradation process to maintain the normal function of cells, and inhibition of lysosomal function with CQ can significantly increase the sensitivity of QBC939 cells to cisplatin. After treated with CQ or 3-MA, the levels of autophagy proteins p62 and LC3-II/I were significantly elevated in QBC939 cells (Fig 5B), indicating that QBC939 cell have a higher flow of autophagy. Compared with HepG2 cells, our results showed that the overall ROS and mtROS levels in QBC939 cells were increased significantly after CQ treatment (Fig 5C–5F), and the mitochondrial membrane potentials were decreased significantly as well (Fig 7D). The results suggested that CQ could induce the increase of ROS level, especially mtROS, in QBC939 cells, which may induce the loss of mitochondrial membrane potentials. MtROS and apoptosis rate were further increased after the treatment of CQ and cisplatin to QBC939 cells, which could be antagonized by Mito-TEMPO (Fig 6A–6C). Therefore, we hypothesized that CQ treatment increased mitochondrial ROS production, induced cell death, and increased the sensitivity of QBC939 cells to cisplatin.

To verify that the CC cell apoptosis induced by CQ was related to the decrease of glucose metabolism-related antioxidant capacity, we examined the influence of CQ and 3-MA on the PPP and found that G6PDH activity, the NADPH/NADP ratio, and GSH/GSSG ratio in QBC939 cells were significantly reduced after CQ treatment (Fig 7A–7C). The decrease in NADPH/NADP and GSH/GSSG ratios may be related to the decrease in G6PDH activity, which may also be a consequence of the increase in cellular ROS induced by the autophagy inhibitor CQ. Compared with 3-MA, CQ has a stronger ability to inhibit PPP (Fig 7A and 7B), which may be related to the inhibition of autophagy by CQ. It is possible that CQ is more involved in the inhibition of autophagy-lysosomal pathway by inhibiting lysosomal function. A recent study found that CQ increases lysosomal membrane permeability, leading to the release of ions such as Fe^2+^ into the cytoplasm. Fe ions combine with intracellular H_2_O_2_ and cause Fenton’s reaction, generating hydroxyl free radicals and elevating the intracellular ROS level [25]. Our results showed that, compared with 3-MA, the amount of hydroxyl free radicals in QBC939 cells was remarkably increased after CQ treatment (Fig 7E). These results suggest that CQ induces a significant increase in the overall ROS level in cells (Fig 5A–5C), possibly due to the disruption of lysosomal membrane permeability and induction of Fenton reaction in the cell.

Therefore, autophagy inhibitor CQ may inhibit the interacting pathway of autophagy and glucose metabolism by blocking the autophagy-lysosomal pathway of cholangiocarcinoma cells, increase hydroxyl free radicals generated in cells, which causes the inhibition of pentose phosphate pathway, decreases cellular antioxidant capacity, and leads to increased MT ROS levels in cells, furthermore, increases the sensitivity of cholangiocarcinoma cells to cisplatin. However, CQ impairs the lysosomal membrane permeability of the cells, and induces the Fenton reaction, increases the generation of hydroxyl radicals in the cells, resulting in an increase in the intracellular overall ROS level. Further study is needed to examine the mechanisms of primary drug resistance inhibition of CQ. However, the current study indicates that inhibition of the autophagy-lysosomal pathway might inhibit the REDOX balance mediated from glucose metabolism in CC cells, increase the level of ROS, induce apoptosis, and increase the sensitivity of cells to cisplatin.

## Abbreviations

2-DG: 2-deoxy-D-glucose
2-NBDG: 2-Deoxy-2-[(7-nitro-2,1,3-benzoxadiazol-4-yl)amino]-D-glucose
3-MA: 3-Methyladenine
CC: cholangiocarcinoma
CQ: chloroquine
DCFH-DA: 2’,7’-dichlorofluorescin diacetate
DHEA: trans-dehydroandrosterone
EGFR: epidermal growth factor receptor
FBS: fetal bovine serum
G6PDH: glucose-6-phosphate dehydrogenase
GSH: glutathione
GSSG: oxidized glutathione
HCC: hepatocellular carcinoma
LC3: microtubule-associated protein 1 light chain 3
Mito-TEMPO: (2-(2,2,6,6-Tetramethylpiperidin-1-oxyl-4-ylamino)-2-oxoethyl)triphenylphosphonium chloride
MM: multiple myeloma
mtROS: mitochondrial ROS
MTT: 3-(4,5-dimethylthiazol-2-yl)-2,5-diphenyltetrazolium bromide
NADP: oxidized form of nicotinamide adenine dinucleotide phosphate
NADPH: nicotinamide adenine dinucleotide phosphate
REDOX: reduction-oxidation
ROS: reactive oxygen species
PBS: phosphate-buffered saline
PPP: pentose-phosphate pathway
